# Simulating unmanned aerial vehicle flight control and collision detection

**DOI:** 10.1186/s42492-019-0014-9

**Published:** 2019-06-21

**Authors:** Mengtian Liu, Meng Gai, Shunnan Lai

**Affiliations:** 1 Peking University Shenzhen Graduate School, Shenzhen, 518055 China; 2 School of EECS, Peking University, Beijing, 100871 China; 3 Beijing Engineering Technology Research Center of Virtual Simulation and Visualization (Peking University), Beijing, 100871 China

**Keywords:** Unmanned aerial vehicle, Proportional-integral-derivative control algorithm, Orientation control, Position control, Grid, k-dimensional tree, Collision detection

## Abstract

An unmanned aerial vehicle (UAV) is a small, fast aircraft with many useful features. It is widely used in military reconnaissance, aerial photography, searches, and other fields; it also has very good practical-application and development prospects. Since the UAV’s flight orientation is easily changeable, its orientation and flight path are difficult to control, leading to its high damage rate. Therefore, UAV flight-control technology has become the focus of attention. This study focuses on simulating a UAV’s flight and orientation control, and detecting collisions between a UAV and objects in a complex virtual environment. The proportional-integral-derivative control algorithm is used to control the orientation and position of the UAV in a virtual environment. A version of the bounding-box method that combines a grid with a k-dimensional tree is adopted in this paper, to improve the system performance and accelerate the collision-detection process. This provides a practical method for future studies on UAV flight position and orientation control, collision detection, etc.

## Background

An unmanned aerial vehicle (UAV), commonly known as a drone, is an aircraft without a human pilot aboard. It can climb, fall, hover, yaw, etc. UAVs are relatively small and convenient to use. UAVs have broad application prospects in military and civilian areas, including intelligence access, target tracking, monitoring, etc. The UAV is an underactuated system [1] that has six degrees-of-freedom (position and orientation) and multiple control inputs (e.g., rotor speed). It also has multivariable, non-linear, and strong coupling characteristics, all of which make its flight-control design very difficult. In UAV simulation systems, the interaction between a UAV and its possibly-complex surrounding environment must be considered; hence, accurate collision detection is another focus. Accurate collision detection can improve the authenticity and reliability of the UAV simulation system, giving the user a better sense of immersion.

In recent years, with the continuous development and improvement of UAV control theory, applying better control algorithms to flight-control systems has become one of the key problems studied by flight-control researchers. Many different control methods have been presented, e.g., backstepping control [2] and chattering-free sliding-mode altitude control [3], which were applied to UAV flight control and achieved good results. Because the backstepping control method had a certain degree of dependence on the model, Farrell et al. [4] first used neural networks to eliminate dynamic modeling errors. Then, they used a backstepping control method to design a four-rotor controller, and achieved better simulation results. In addition, special tools, e.g., MATLAB/Simulink [5], have also contributed much to this field.

Scholars in the collision-detection field have conducted extensive research and presented efficient detection methods in recent years. These methods can be divided into two phases: spatial-decomposition methods and hierarchical bounding-box methods, they are used as much as possible to reduce the number of collision tests. Spatial decomposition provides broad-phase processing by dividing space into regions, and testing whether objects overlap the same region. It mainly includes three types of spatial partitioning: grids, trees [6], and spatial sorting. The problem with spatial partitioning is determining when to stop dividing the space cells and setting the cell sizes. Turk et al. [7] first proposed using spatial hash tables with uniform partitions to improve the query speed. However, this method is more suitable for cases where the objects’ positions are dispersed. In the worst case, i.e., the objects’ positions are concentrated, it has an O(n^2^) time complexity. The basic idea of the hierarchical bounding-box method is to use simple geometry to progressively break up the surrounding model. Intersection tests are then used to quickly eliminate disjoint areas, thereby reducing the overall number of tests. Many bounding-box algorithms have been presented: Sphere [8], axis-aligned bounding boxes [9], and oriented bounding boxes [10].

But these methods mentioned above do not have special optimizations for certain scenarios. In this paper, we proposed a hybrid method combining spatial decomposition method with hierarchical method to detect collision. As a result, we can catch small details with high speed.

## Methods

### UAV orientation and position control

**Fig. 1:**
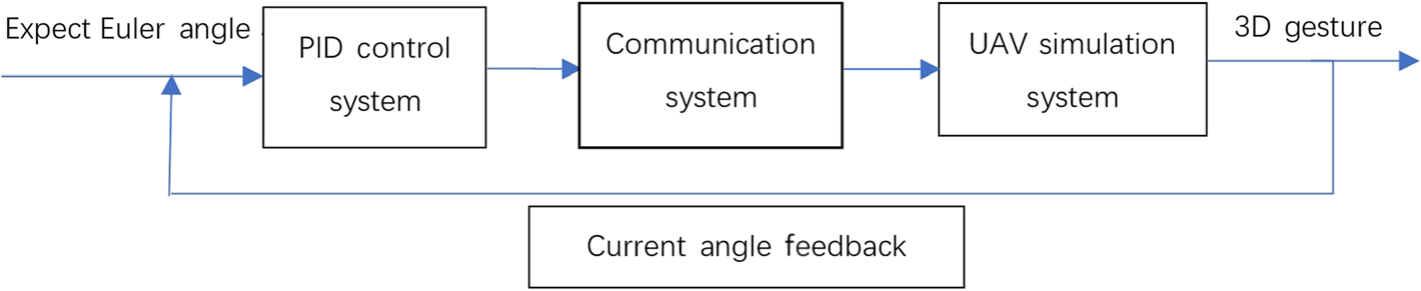
Proportional-integral-derivative control schematic diagram

Orientation control is the premise for realizing many complex UAV functions; i.e., it is the core of UAV control. This study adopts the proportional-integral-derivative (PID) control method to control the UAV because of the feasibility and superiority of its attitude control algorithm. Fig. 1 shows a block diagram of the PID control method. The expected angle is the angle of the remote control that controls the UAV. The current angle is measured by the UAV simulation system, where the angle refers to the Euler angle (pitch, yaw, and rotation angles). In the PID control-calculation process, these three angles are independent of each other. It’s calculation process can be simplified as follows:

1. Calculate the axial deviation (deviation = target desired angle - measured angle);
2. Calculate the proportional term (ratio coefficient P * deviation), integral term (integral coefficient I * angular rate), and differential term (differential coefficient D * angular rate);
3. Sum the resulting outputs (total direction control = proportional item output + micrometer output + integral item output).

The position control guides the UAV along the specified trajectory or in accordance with the designated location hover. The position control in this study is divided into vertical and horizontal position controls. The vertical control calculates the difference between the desired height and the actual height as the desired climb rate, and then uses the PID control algorithm to obtain the UAV’s height. The horizontal-position PID control uses the difference between the expected position and the actual location as the required distance to calculate the desired speed, and then uses the PID control algorithm to obtain the UAV’s position.

### Spatial partition using a grid and a k-dimensional tree

**Fig. 2:**
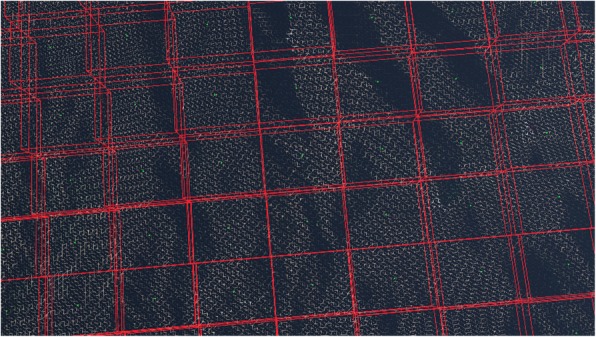
Meshing in a virtual simulation system

The UAV’s collisions with objects (e.g., trees, ground, and towers) should be considered when it is flying in a simulated natural environment. We divided the colliders into two categories, static and dynamic. Generally speaking, dynamic colliders are much rarer than static ones, so they can be filtered individually according to their distance from the UAV. Thus, this paper mainly discusses collisions between UAVs and static colliders. In general, complex environments have a large number of colliders. If we check for collisions with all of the colliders, the algorithm will have a high time cost, and its real-time performance cannot be guaranteed. For a UAV flying in a virtual simulation environment with a large number of trees, grass, etc., it is important to organize the objects that may collide with the UAV. Because we know beforehand the positions of all possible colliders, we can use uniform-grid technology to divide the simulated natural environment into a number of equal-sized regions or grid cells (Fig. 2). The whole process can be summarized as the following steps:

1. For the area where the UAV is located, select the area size (all areas cover all virtual objects);
2. The area obtained in the first step is divided into grid cells of appropriate size by the equalization grid technique;
3. The objects in each grid cell are organized into a k-d tree, and each grid cell holds the root node pointer of the k-dimensional tree (k-d tree) it owns.

**Fig. 3:**
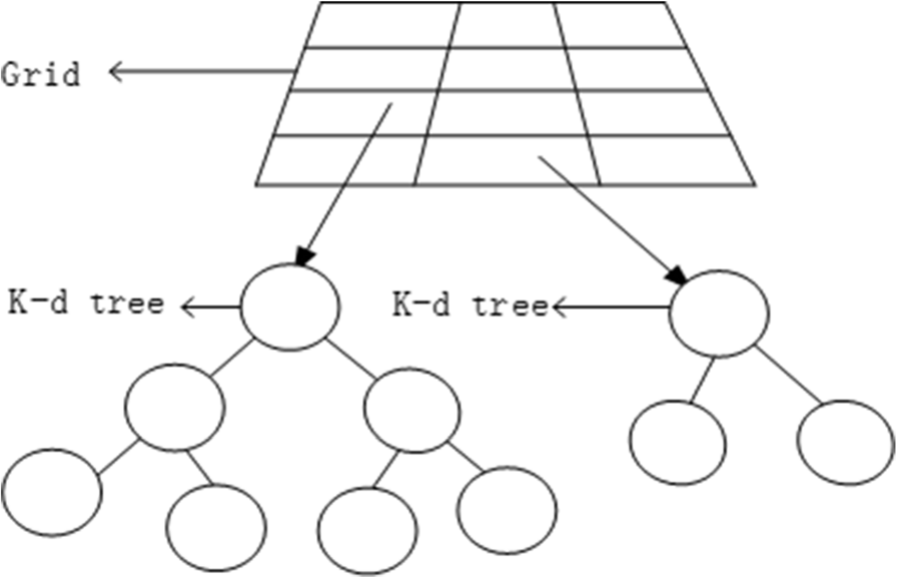
The structure of sectional static colliders

At last, we can get the objects in every grid cell are organized by k-d tree structure (Fig. 3). Combining grid technology and k-d tree technology, we transform the problem of detecting a collision between the UAV and a surrounding object (e.g., tree or tower) into finding a possible collision object located on the k-d tree pointed to in the grid at the UAV’s current location. We then find the possible collision object that is nearest the UAV. This is similar to the k-d tree’s nearest neighbor search problem. Many efficient algorithms have been presented for finding the node closest to the UAV’s location in a given k-d tree structure. Precise collision detection is very time-consuming. Through a combination of grid technology and a k-d tree, we only need to accurately detect a collision between the UAV and a predetermined collision object. This greatly reduces the time required for collision detection.

### Collision detection

First, we should address the problem of creating a hierarchical bounding box. There are three primary construction-method categories: top-down, bottom-up, and insertion. Among them, top-down methods partition the input into two or more subsets, bound them in the chosen bounding volume, and then recurse over the bounded subsets. Top-down methods are by far the most popular, owing to their ease of implementation. Insertion methods build the hierarchical bounding box incrementally by inserting objects one at a time into the tree. Bottom-up (or agglomerative) methods start with the leaves of the tree as the input set, and then group two or more of them to form a new (internal) node. Bottom-up methods take longer to construct than top-down methods, but usually produce better trees [11].

**Fig. 4:**
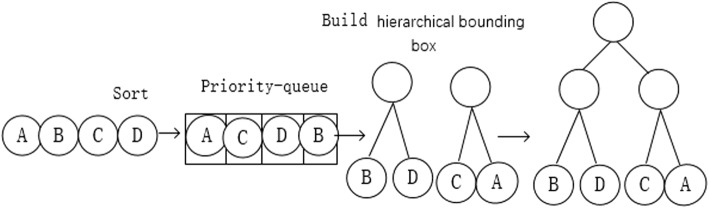
The construction of hierarchical bounding box tree (take binary tree as example, the circle in the figure stands for primitive that make up the collider)

**Fig. 5:**
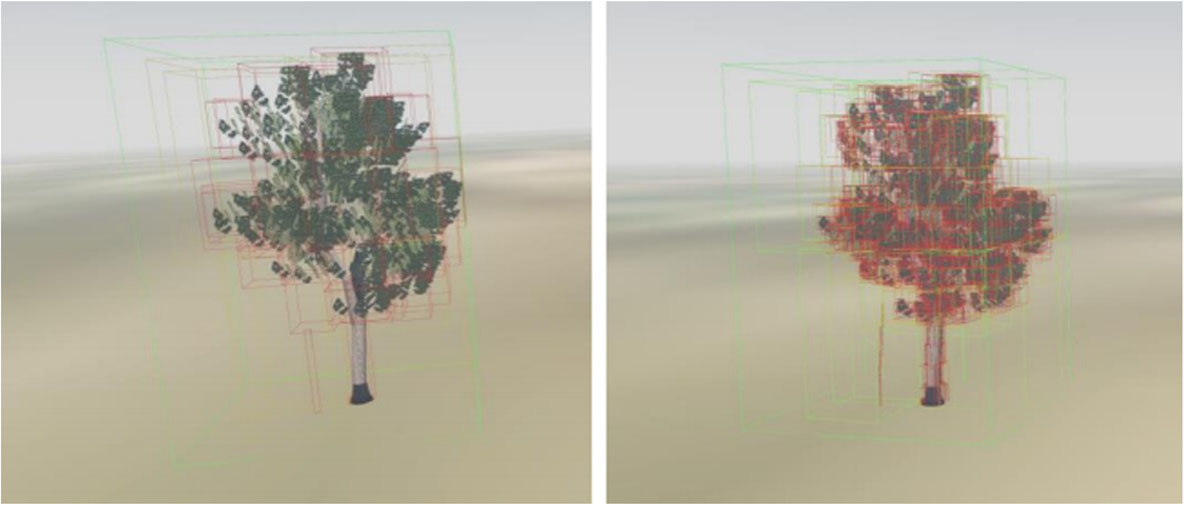
Hierarchical box tree with different levels (number of layers on the left is 3, on the right is 5)

In this study, we chose an improved bottom-up method to create the hierarchical bounding box. Conventional bottom-up methods for finding which two nodes to merge involve examining all possible pairs, computing their bounding volume, and selecting the pair with the smallest bounding volume. This requires O(n^2^) time. Moreover, it must be repeated n-1 times to form a full tree; thus, the total construction time becomes O(n^3^). We can sort the leaf nodes according to the bounding box’s volume, and then use a priority-queue structure to store them. It is similar to creating a Huffman tree structure, and the total construction time is reduced to O(n^2^ log n). The process of creating the hierarchical bounding box is depicted in Fig. 4. By adopting this method, we can define the maximum depth of the collision tree, which is equivalent to the number of layers in the bounding box. Fig. 5 shows the bounding-box levels for a tree model; green indicates a coarse bounding box, and red indicates a fine bounding box. After constructing the hierarchical bounding boxes for the UAV and the possible collider, we can execute the collision-detection algorithm. The hierarchical bounding-box collision-detection algorithm is described as follows, where a&&b indicates a precise collision detected between hierarchical bounding boxes a and b.

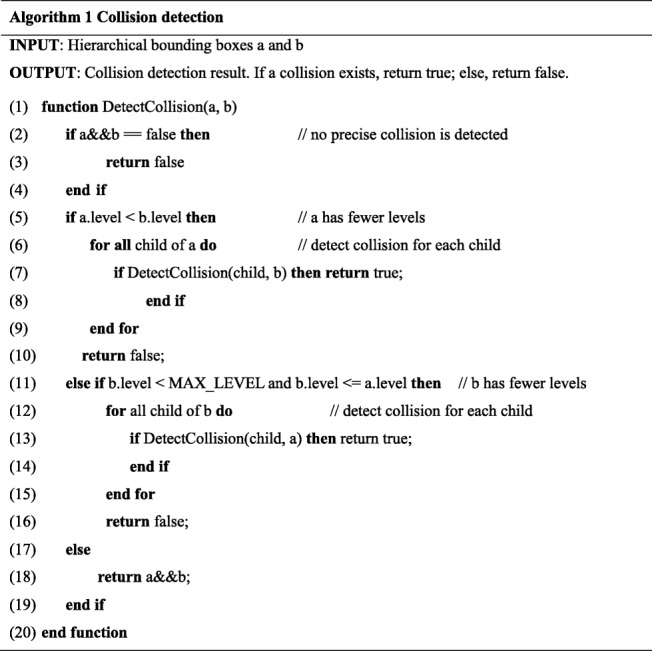



## Results

**Fig. 6:**
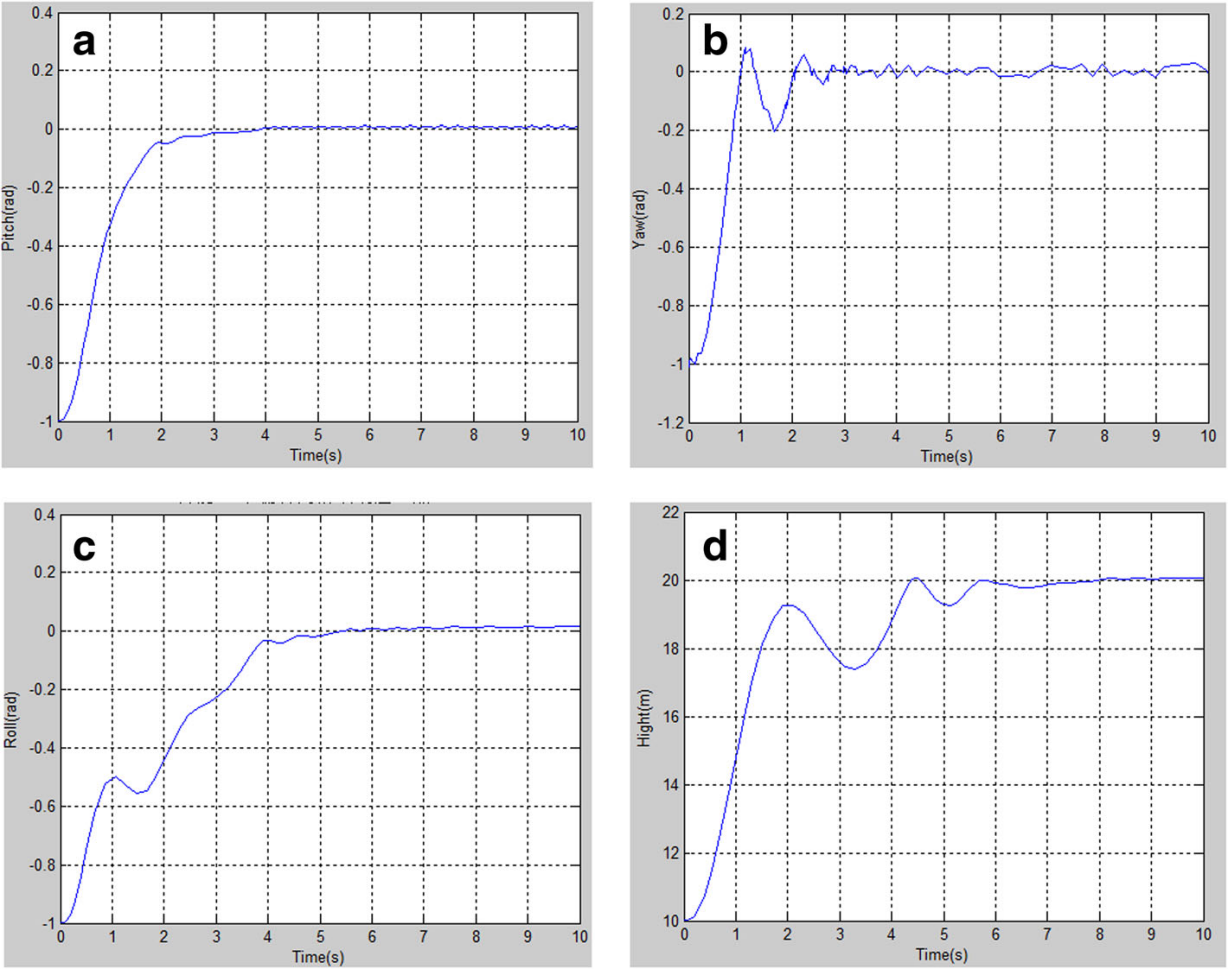
The simulation results. **a** Pitch angle simulation; **b** Yaw angle simulation; **c** Roll angle simulation; **d** Height simulation

The PID parameter values selected in this paper are shown in Table 1. We used MATLAB software to simulate the PID control algorithm used in this paper, to verify its feasibility. The simulation results show the UAV’s roll angle (Fig. 6a), pitch angle (Fig. 6b), and yaw angle (Fig. 6c), which are initially located at −1 rad; it eventually reaches a hover effect in the simulation curve. The hover simulation results indicate that the pitch and yaw angles reached 0 rad in a short period of time, and the roll angle reached 0 rad after 4 seconds. The yaw angle had an obvious fluctuation, and the other two angles had very small fluctuation ranges. Similarly, we can see in Fig. 6d that the UAV’s height reached about 20 m in 5 seconds and keep stable, starting from an initial value of about 10 m. The simulation results show that the PID control algorithm adopted in this paper performed well.

**Table 1:** Proportional-integral-derivative parameter

Gesture of unmanned aerial vehicle	Proportional P	Integral I	Differential D
Pitch angle	2.7	0.03	0.75
Roll angle	2.8	0.04	0.80
Yaw angle	4.4	0.06	1.50
Height	3.0	0.08	1.25

**Fig. 7:**
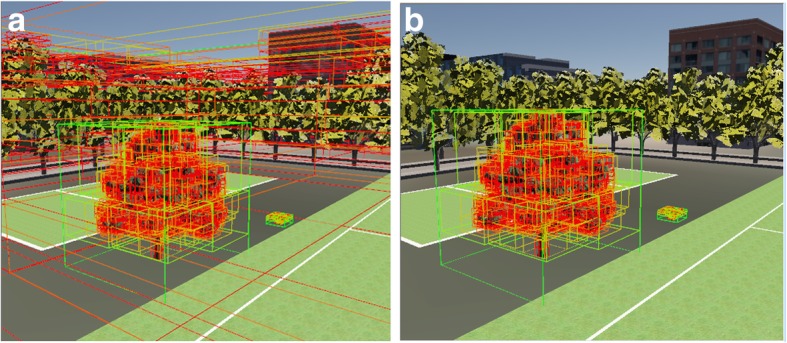
The structures of possible colliders. **a** Results adopted by traditional collision detection; **b** Results adopted by our algorithm

We tested our algorithm on a personal computer with a 3.20 GHz Core™ i5–4570 CPU, an NVIDIA GeForce GTX 1060 graphics card, and 8 GB of memory. Fig. 7a shows the results of a traditional hierarchical bounding-box technique without partitioning technology, used to detect collisions between a UAV and objects in a complex virtual environment. Fig. 7b shows the results of our method. Our algorithm only calculates the hierarchical bounding boxes of the UAV and its nearest possible collider.

**Fig. 8:**
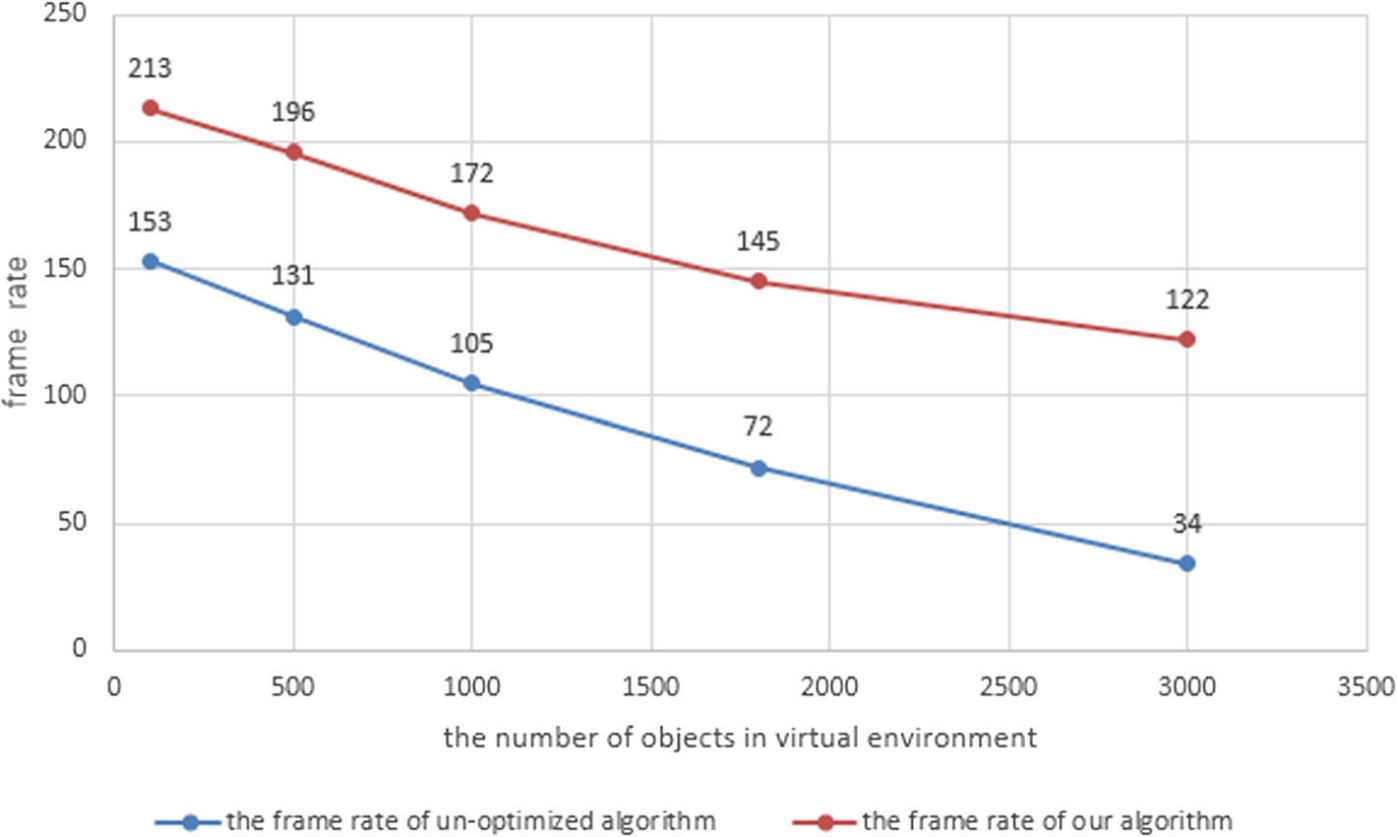
The frame rate of our algorithm compared with un-optimized algorithm

**Fig. 9:**
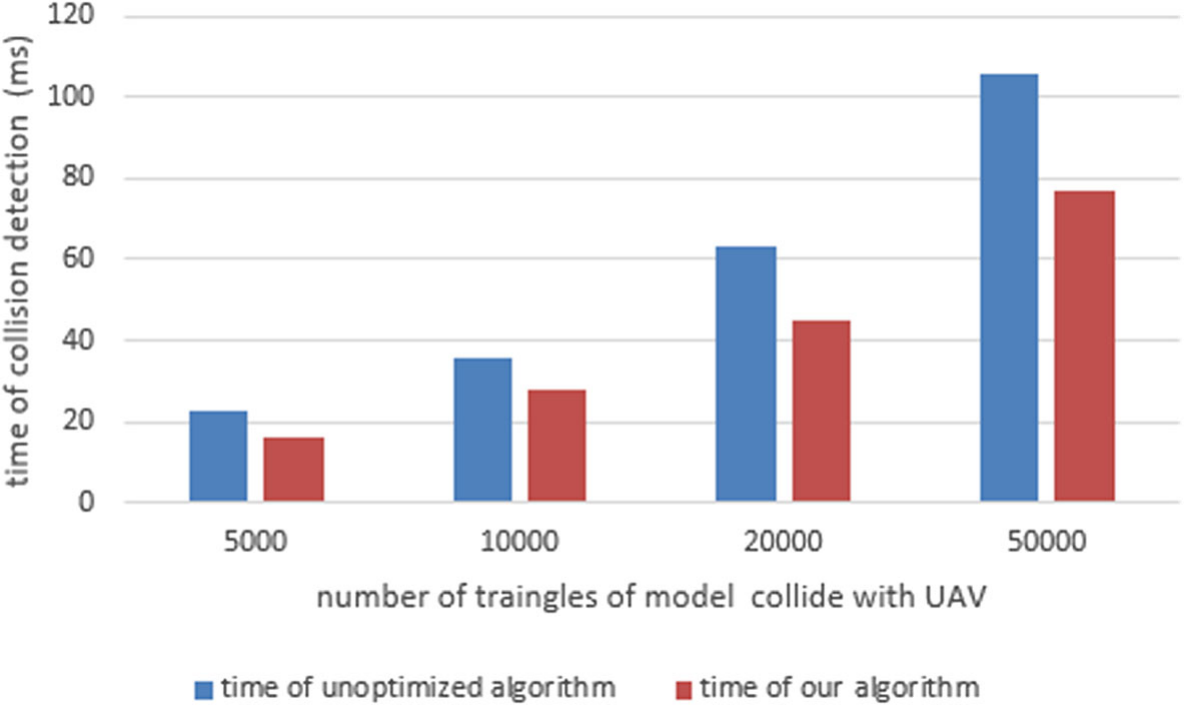
The time of collision detection of our algorithm compared with un-optimized algorithm

The UAV model adopted in this paper contains about 15000 triangles. We chose a complex simulated environment containing 100, 500, 1000, 1800, and 3000 models of trees, houses, etc., in turn. Fig. 8 shows the frame-rate results of a simulation system adopting our algorithm and one using an un-optimized algorithm. As seen in the Fig. 9, the traditional collision-detection algorithm must maintain the hierarchical bounding-box construction for all possible colliders located near the UAV in every frame of the simulation. This wastes time and reduces the system performance. Our algorithm only needs to maintain the hierarchical bounding-box construction of the UAV and its nearest possible collider. From Fig. 7a, we can observe that, as the number of possible colliders in the virtual environment increases, our algorithm becomes increasingly superior to the un-optimized algorithm, in terms of the frame rate of the UAV simulation system. Fig. 9 shows the time needed to detect a collision between the UAV and different colliders. The results show that, by optimizing the construction of the hierarchical bounding-box tree, and choosing appropriate collision-tree layers, our algorithm is more efficient than the un-optimized algorithm. Our algorithm will be even more efficient when the possible collider has more patches.

## Discussion and conclusions

**Fig. 10:**
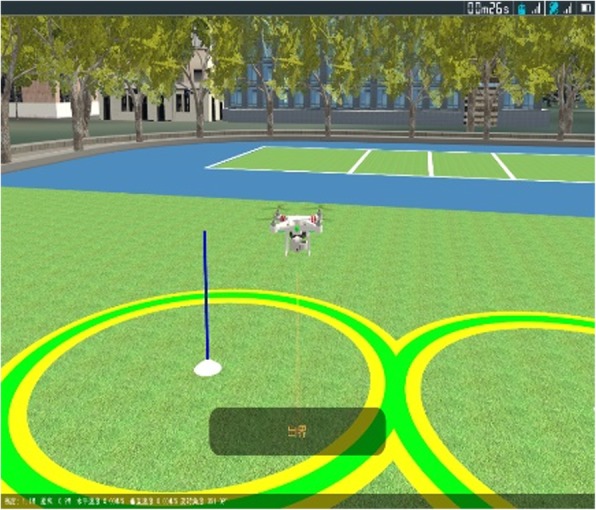
Unmanned aerial vehicle flying around the rings

**Fig. 11:**
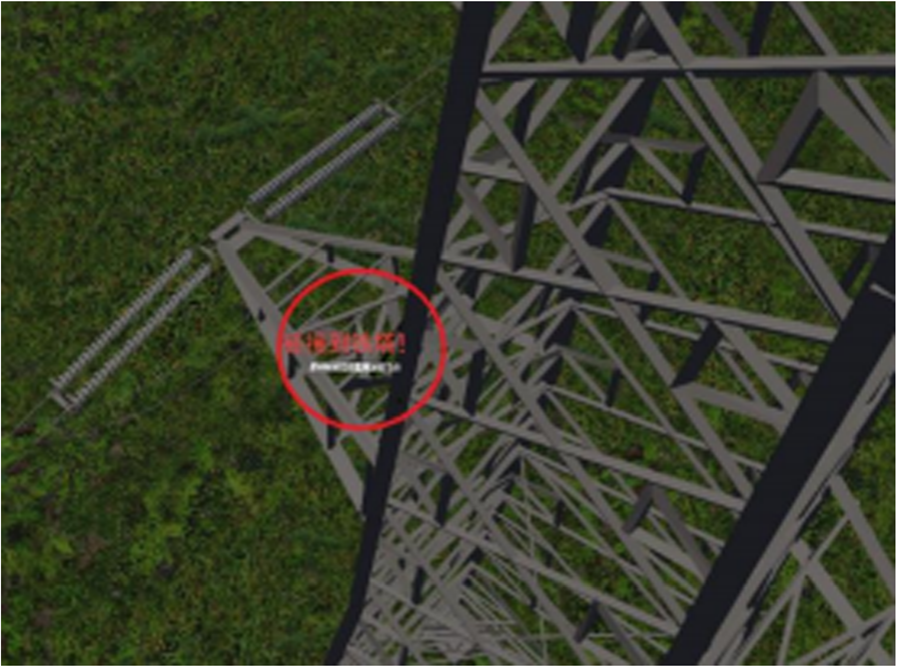
Unmanned aerial vehicle collides with the tower

We used MATLAB simulation experiments to verify the PID algorithm for the UAV’s attitude and position-control effectiveness. Using the PID control algorithm and collision-detection algorithm described in this paper, we created a simulation system that can control the position and orientation of a UAV. For example, it can make the UAV fly along an established trajectory, or along a path consisting of two rings, as shown in Fig. 10. In this paper, we proposed a method to improve the accuracy of the bounding-box method by combining grid technology with a k-d tree. This was applied to our UAV simulation system to increase the system performance and reduce the time required for collision detection. We can effectively detect collisions that occur between the UAV and objects, e.g., trees, towers, and the ground, in the virtual environment (Fig. 11).

Although we used the classical PID control algorithm in our flight-control system, PID is not robust in terms of parameter uncertainties, and it is difficult to tune its parameters for unstable systems. Recent studies have shown that we can build a stable and precise flight-control system by combining it with a linear quadratic regulator [12]. As future work, we plan to further improve the control-system software functions and increase the flight-control system autonomy, e.g., automatic obstacle avoidance, automatic following, and independent completion of scheduled complex functions.
